# Multifunctional 3D printing of heterogeneous hydrogel structures

**DOI:** 10.1038/srep33178

**Published:** 2016-09-15

**Authors:** Ali Nadernezhad, Navid Khani, Gözde Akdeniz Skvortsov, Burak Toprakhisar, Ezgi Bakirci, Yusuf Menceloglu, Serkan Unal, Bahattin Koc

**Affiliations:** 1 Faculty of Engineering and Natural Sciences, Sabanci University, Orhanli-Tuzla, Istanbul, 34956, Turkey; 2 3D Bioprinting Laboratory, Sabanci University Nanotechnology Research and Application Center, Orhanli-Tuzla, Istanbul, 34956, Turkey; 3 Sabanci University Nanotechnology Research and Application Center, Orhanli-Tuzla, Istanbul, 34956, Turkey

## Abstract

Multimaterial additive manufacturing or three-dimensional (3D) printing of hydrogel structures provides the opportunity to engineer geometrically dependent functionalities. However, current fabrication methods are mostly limited to one type of material or only provide one type of functionality. In this paper, we report a novel method of multimaterial deposition of hydrogel structures based on an aspiration-on-demand protocol, in which the constitutive multimaterial segments of extruded filaments were first assembled in liquid state by sequential aspiration of inks into a glass capillary, followed by *in situ* gel formation. We printed different patterned objects with varying chemical, electrical, mechanical, and biological properties by tuning process and material related parameters, to demonstrate the abilities of this method in producing heterogeneous and multi-functional hydrogel structures. Our results show the potential of proposed method in producing heterogeneous objects with spatially controlled functionalities while preserving structural integrity at the switching interface between different segments. We anticipate that this method would introduce new opportunities in multimaterial additive manufacturing of hydrogels for diverse applications such as biosensors, flexible electronics, tissue engineering and organ printing.

Progress in additive manufacturing demands substantial improvements in deposition techniques while addressing current challenges in controlling structural features in macro, as well as micro scales. One of the major challenges is to fabricate heterogeneous multimaterial constructs with high dimensional precision with the lowest possible defects at the interfacial sections at the same time. Recent studies on multimaterial 3D printing using direct writing or fused deposition modeling mostly include use of multi nozzle deposition systems in fabrication of functional devices^1^,^2^,^3^, and scaffold based^4^,^5^,^6^,^7^ and scaffold free^4^,^8^,^9^ tissue engineering approaches. Moreover, studies have been reported on multimaterial 3D printing by using single nozzle^10^,^11^,^12^ designs, employing a range of different inks including ceramic pastes, viscoelastic polymers and hydrogels. However, there are several challenges related to process parameters and material behavior in those current multi-material 3D printing strategies. For multi-nozzle based approaches, switching between multiple nozzles might introduce dimensional inaccuracies and discontinuities between material patterns due to the possibility of misalignment of different nozzles and the possible defects as a result of start and stop of ink flow. On the other hand, controlling the switching interface in multimaterial deposition of inks determines the final precision of the process and current methods of deposition based on instant material-switching protocols in single nozzle designs often produce transient regions at the interface composed of an uncontrolled mixture of sequenced inks. Approaches in controlling deposition parameters of multimaterial 3D printing by a single nozzle significantly affect the performance and feasibility of design. Recent study by Hardin *et al*.^11^ is an excellent example in which they employed a well-tuned set of parameters by considering ink properties to efficiently control the interface between neighboring segments. However, their approach is limited by ink’s viscosity and does not seem to be applicable in the case of low viscous materials like hydrogels. In addition, switching or mixing nozzle based approaches could produce compositional inhomogeneities induced by formation of dead volumes. Ink properties play a crucial role in controlling printing parameters and the suitability of a printing method for certain type(s) of materials is eventually determined by ink’s properties^13^.

In this paper, we report a novel and versatile technique for multimaterial 3D printing of hydrogels, with the ability to deposit complex patterns and produce diverse functionalities in printed constructs. A range of model thermally cross-linked hydrogels based on agarose with different concentrations, compositions and encapsulated cell densities were printed to demonstrate the capabilities of our method. We have employed a sequential aspiration-on-demand method which is determined automatically by the developed Computer-Aided Design (CAD) based algorithm. In this approach, sequential aspiration volume of multimaterial segements and printing path plan are determined directly from computer models. The calculated constitutive multimaterial segments of each printed filament were then assembled in liquid state by sequential aspiration of inks into a glass capillary followed by *in situ* gel formation upon decreasing the temperature and printing based on the calculated paths. The physical properties of inks were considered in tuning process parameters such as process temperature, interfacial features in switching segments and functionality, stability, and integrity of the printed objects.

## Results

Different agarose ink concentrations and compositions were prepared and used in printing of patterned constructs (Table 1). Agarose hydrogels with different concentrations were used as the primary inks. Altering the composition was demonstrated by using single-walled carbon nanotubes (SWCNT) embedded in agarose hydrogels. By inclusion of NIH 3T3 cells inside agarose ink, which is referred to as cell laden bio ink, the function of agarose ink was modified from originally being as the support structure to be as the cell carrier.

### Deposition of a complex patterned object by altering ink composition

**Figure 1:**
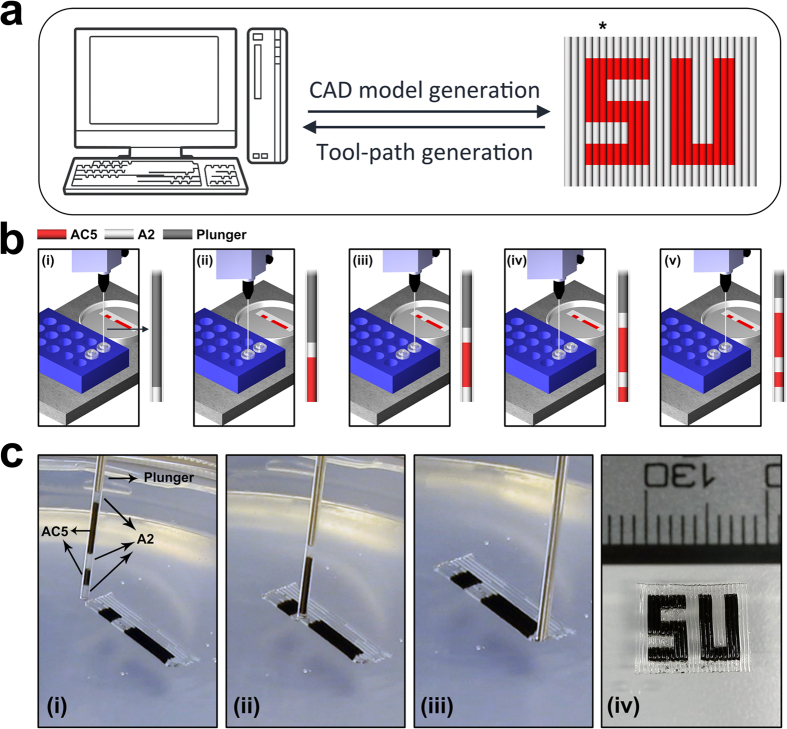
Schematic representation of printing process for deposition of a patterned structure. (**a**) CAD model is generated in an appropriate software and tool-path is developed by considering the material switching orders. (**b**) Aspiration orders are assigned to the computer-controlled aspiration/deposition system which is installed on a three-axis platform. Step-by-step aspiration of a filament is shown with respect to its build sequence defined in the CAD model (marked by asterisk in panel (a)). (**c**) (i–iii) real-time images captured during printing of a complex pattern. Aspirated filament in (i) consists of five different ink segments. (iv) Printed SU (Sabanci University) logo which includes segments of A2 (transparent) and AC5 (black) inks.

A schematic representation of the printing process and a deposited patterned structure are shown in Fig. 1. In a typical printing session, path plans are generated based on the desired printed geometry and the segmented models are used to assign the aspiration sequences automatically by the developed algorithm. Tool-path is generated according to the modeled structure and automatically translated to machine language with the aid of the developed algorithm. Switching between different materials is precisely controlled by considering two parameters including materials reservoir temperature and interface between two sequentially aspirated materials. The former directly affects inks viscosity while the latter determines the smoothness of transient section between two neighboring segments and geometrical integrity in deposited filaments.

**Figure 2:**
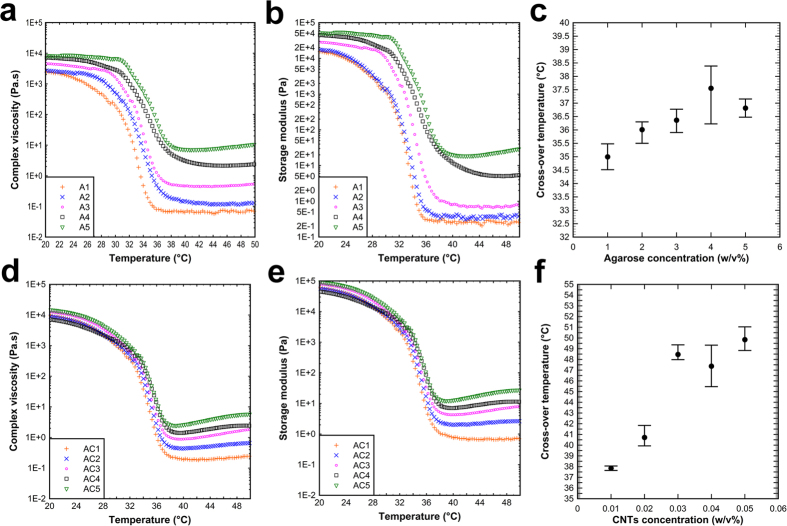
Oscillatory rheometry data representing the effect of agarose and SWCNT concentrations on physical properties of inks. Viscosity of inks for (**a**) agarose and (**d**) agarose-SWCNT hydrogels as a function of temperature. Storage modulus of inks based on (**b**) agarose and (**e**) agarose-SWCNT as a function of temperature. Cross-over temperatures (G′ = G′′) for (**c**) agarose and (**f**) agarose-SWCNT inks as a function of ink composition. Graph (**f**) plots the cross-over temperatures of AC1 to AC5 inks in which the concentration of agarose was kept constant at 2 w/v%.

Since agarose based inks are thermally cross-linked hydrogels, viscosity was a function of temperature and gelation occurred as an irreversible thermal crosslinking process, except for AS3 and A-NIH inks in which the gelation was thermally reversible. Temperature plays a crucial role in determination of ink viscosity, and affects process parameters such as aspiration temperature and the timing between aspirations sequences, the later was controlled by printer head speed. Printer head speed will affect the viscosity of already aspirated ink since it determines the time duration wherein the glass capillary is out of ink’s reservoir and is exposed to environment temperature. A simplified transient thermal analysis was performed by using ANSYS software to roughly predict the temperature profile in an already-aspirated ink while the capillary is exposed to environmental temperature during ink switching (Supplementary Movie 1). Figure 2 demonstrates the dependence of inks viscosity and storage modulus of inks on temperature.

Referring to viscosity-temperature profiles (Fig. 2a,d), temperature of inks reservoir was set to 50 °C at which all inks showed liquid like behavior and viscosities were low enough to ease the aspiration. Cross-over points are plotted as a function of agarose and SWCNT concentrations in Fig. 2c,f. Above cross-over temperatures, the contribution of liquid-like behavior on the rheology of mixtures was dominant and cross-over points can be considered as the turning point below which inks tend to show more elastic behavior. However, cross-over points in AC3 to AC5 inks increased dramatically compared to the same values in AC1 and AC2 inks. This increase in cross-over temperature can be attributed to the formation of SWCNT network in the mixture, indicating high performance of dispersion step during ink processing (Supplementary Fig. 1). Trends in viscosity profiles of AC3 to AC5 inks during cooling (the plateau region before drastic increase in viscosity) seemed to be similar to the other inks although the cross-over points shifted to higher temperatures. The consistency in viscosity profiles of hydrogel-SWCNT inks with the base agarose ink (A2) indicates the printability of their combination in the same processing temperature, regardless of the more elastic contribution in corresponding inks at a temperature span of about 10 degrees. Rheological data together with results of heat flow simulation during ink switching were used to adjust the printer head speed. Thermal simulations showed that the temperature drop during ink switching in previously aspirated ink can be maintained within an acceptable range at which gelation would not occur, and change in viscosity of inks is negligible.

**Figure 3:**
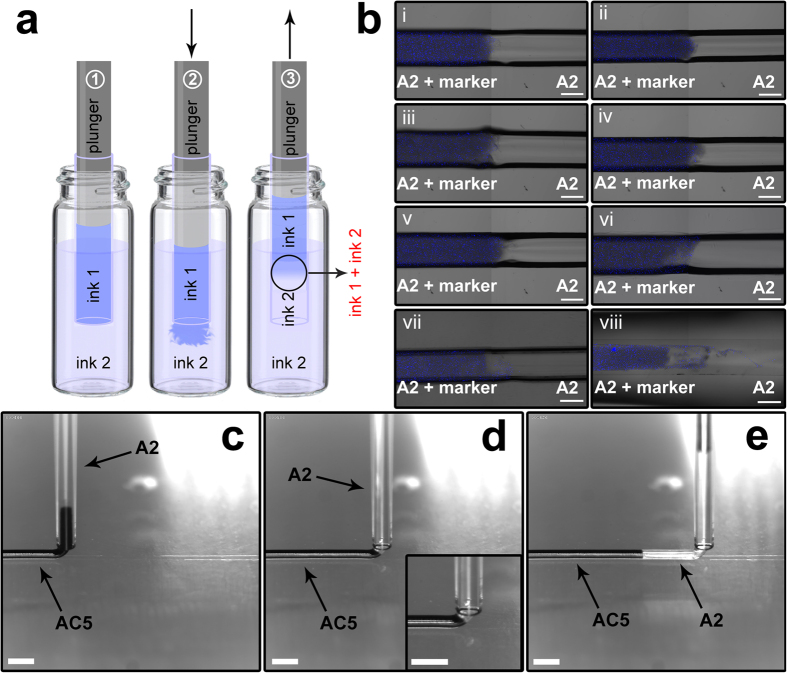
Interface between two segments of different inks in a single filament. (**a**) Schematic representation of aspiration sequences in a typical printing practice. Displacement of plunger in stage (2) can be controlled by tool-path and varied from zero to 500 μm. Plunger speed was set to 50 mm/min to minimize the turbulent ink flow. (**b**) (i–vi) Confocal microscopy images of filaments deposited by setting the plunger displacement in (**a**2) to values of zero to 500 μm with 100 μm increments, respectively; scale bars, 250 μm. (vii,viii) Effect of plunger speed in formation of turbulent ink flow by setting plunger speed at 100 mm/min while the plunger displacement in (**a**2) was set to zero and 500 μm, respectively; scale bars, 250 μm. (i–viii) Fluorescent micro-beads were used to mark the segments in each filament and A2 ink without fluorescent markers was aspirated first. (**c**–**e**) Images taken by high speed camera during deposition of one single filament composed of A2 and AC5 inks while the plunger displacement in (**a**2) was set to zero; scale bars, 1 mm. Magnified image of capillary tip and extruded filament in inset (**e**) shows the high stability of interface under high applied stresses during deposition.

In addition to process temperature, the plunger displacement during aspiration of multi-ink filaments should be handled carefully to make sure that deposited filament has high degree of structural and geometrical integrity at the interface between two neighboring segments where ink switching occurs. To investigate the quality of segments’ assembly in aspiration of each filament, we introduced a parameter called the transition zone length (TZL). A transition zone between two sequenced segments is produced by extrusion of a designated length of previously aspirated ink inside the next ink’s reservoir, just before commencing the next aspiration (Fig. 3a). This will result in formation of a tiny volume of two inks mixture, which can be controlled by the plunger displacement. Increase in TZL was observed by increasing the volume of extruded ink inside reservoir before the next aspiration (Fig. 3b(i–vi)). Extended TZL values will result in a smoother transition between two neighboring segments, and depending on desired output property (chemical, electrical, mechanical, or biological properties), TZL can be roughly controlled. However, due to the risk of contamination in ink reservoir and non-homogeneous nature of mixture at the interface, TZL is practically limited to several hundreds of micrometers. It should be noted that plunger speed during aspiration must be low enough to prevent from formation of turbulent ink flow, and at the same time, it should be high enough to keep the processing time reasonable. Figure 3b(vii,viii) shows the effect of increasing plunger speed from 50 to 100 mm/min on formation of non-homogeneous mixture of two inks at the interface. Even by setting the plunger displacement at second stage of aspiration to zero (Fig. 3a), formation of a turbulent ink flow caused extension of TZL at the interface (Fig. 3b(vii)). A combination of large plunger displacement before aspiration and high aspiration rate resulted in generation of significantly non-homogeneous transition zone in orders of millimeters (Fig. 3b(viii)). Images taken by high-speed camera during deposition of a single segmented filament (Fig. 3c–e) clearly demonstrate that even by setting the plunger displacement at stage 2 (Fig. 3a) to zero, geometrical and structural integrity of filaments were preserved precisely at the switching interface and in the meantime, switching interface withstands the high shear and normal stresses at the tip of capillary during deposition (Supplementary Movie 2). This observation indicates the independence of filament geometrical integrity at switching interface from TZL, as far as two neighboring segments have been aspirated while they are still in the liquid (low viscosity) state.

### Deposition of hydrogel inks with geometrically patterned properties

To demonstrate the potential of our method in producing patterned structures, several patterns were generated by altering chemical, mechanical, electrical, and biological properties of deposited structures. Changing the chemical composition in a controlled manner is shown in Fig. 1c. Printed SU (Sabanci University) logo comprises several filaments each was composed of one or more segments of A2 and/or AC5 inks. Accurate translation of CAD geometry into machine codes and adaptation of process parameters to inks’ physical properties resulted in obtaining near net shape object which is in good match with designed CAD model. Reliability of our method in deposition of complex patterns using multi-inks with different compositions could be realized by comparing deposited object with starting CAD model (Fig. 1a,c).

**Figure 4:**
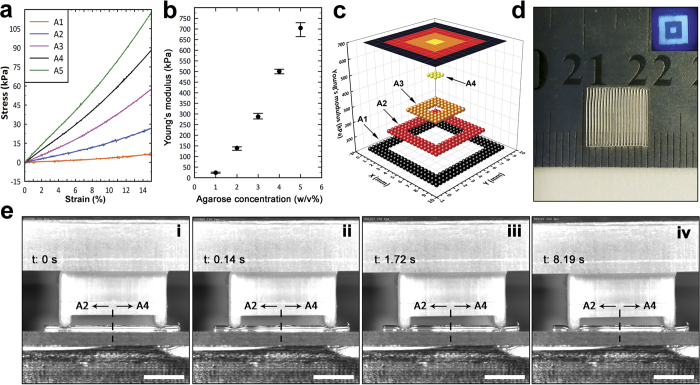
Deposition of agarose with patterned stiffness. (**a**) Stress-strain plots of agarose inks including A1 to A5 under compression. Confined-strain condition was applied during force measurement to prevent from sample breakdown. (**b**) Young’s modulus values for A1 to A5 inks extracted from small-strain (0 to 4%) portion of corresponding stress-strain plots. Hook’s law and the governing equation of linear elasticity (σ = Εε) were used to describe the force-displacement behavior. (**c**) Visualization of patterned stiffness in object (**d**) by plotting Young’s modulus values across different segments of sample. Young’ modulus varied from 23.6±2.0 kPa in the outmost segment to 500.4±7.7 kPa in the square at the center. (**d**) A sample printed by using A1 to A4 inks with a nested-squares pattern. Inset image shows different segments within the sample stained with fluorescent microbeads. (**e**) Timed sequence images during constant loading on printed filaments composed of A2 and A4 segments. Images were taken by high speed camera during constant loading by a free “Pi (Π)” shape bar, while (i–iv) correspond to the moment Pi bar touched the filaments as t = 0 s, release of the bar, and its further penetration downwards due to its own weight, respectively; scale bars, 3 mm.

Controlling over stiffness of printed objects is shown in Fig. 4. To demonstrate the ability to pattern the stiffness in printed constructs, hydrogel inks were first mechanically characterized using compression test to identify their stress-strain behavior. The response to applied normal force in hydrogel inks is demonstrated in stress-strain plots in Fig. 4a. Two regions of linear elastic and non-linear response to applied stress were observed and the maximum stress in strain-confined tests increased significantly by increasing agarose concentration from about 7 kPa to more than 117 kPa. Elastic moduli of inks were determined from small strain portion of stress-strain plots (up to 4% strain) in which samples showed linear elastic properties and they could be described by Hook’s law^14^. Values of Young’s moduli as a function of agarose concentration were plotted in Fig. 4b. Broadness in the range of stiffness values of inks with different agarose concentrations was employed to deposit objects with patterned stiffness properties. A sample pattern of nested squares was printed by using four inks including A1 to A4 in which the concentration of agarose varied over 1 to 4 w/v% (Fig. 4d). Depending on the position of each constitutive hydrogel filament in printed object, each filament was composed of one or more segments. Maximum number of assembled segments was in the case of three filaments passing the center of square, where they were composed of seven different segments of four different inks; meaning that there were up to six times of switching between different inks during aspiration of each of those filament. To visualize the deposited stiffness pattern clearly, Young’s modulus values of different regions of sample are plotted in Fig. 4c as well as a contour merging all segments. Young’ modulus changed from 23.6 ± 2.0 kPa in the outmost segment to 500.4 ± 7.7 kPa in the square at the center. To demonstrate how the mechanical properties of different segments contributed to the overall mechanical response of a printed object, a constant load was applied on a set of printed filaments consisted of A2 and A4 inks by using a free “Pi (Π)” shape bar, as is shown in Fig. 4e. The timed sequence of images during loading was captured by high speed camera, started by the moment at which the Pi bar touched the filaments (Fig. 4e(i)) followed by its release (Fig. 4e(ii–iv)) (see also Supplementary Movie 3). Figure 4e also shows the possibility of tuning mechanical functionalities of printed object to respond differently to external stimuli.

**Figure 5:**
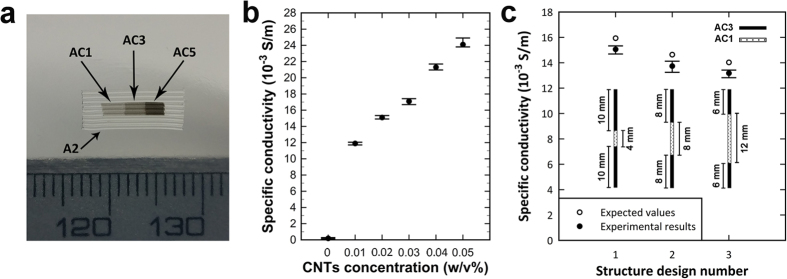
Patterning electrical conductivity in deposited hydrogel structures. (**a**) Printed object with patterned electrical properties by using combination of A2, AC1, AC3 and AC5 inks. Electrical conductivity increased from AC1 to AC5 segments while the matrix (A2) possessed very low conductivity value. (**b**) A plot showing the increase in the specific conductivity of inks by changing the SWCNT concentration. (**c**) Values of specific conductivity for three different structure designs. Each data point indicates the average specific conductivity for single filaments printed with respect to the corresponding design of segments. Expected specific conductivity values were calculated by applying the series circuit rules, using the data presented in (**b**) for each constitutive segment.

SWCNT embedded hydrogel inks were used to selectively pattern the electrical conductivity of deposited structures. Specific conductivity of filaments composed of A2 (SWCNT concentration equals to zero) and AC1 to AC5 inks are plotted in Fig 5b. Incorporation of SWCNTs in agarose enhanced the conductivity although this enhancement was not much significant compared to a previous study on agarose/CNT hydrogel nanocomposites^15^, since AC1 to AC5 inks had considerably low SWCNT concentrations which were not enough to induce noteworthy effects on electrical properties of matrix. However, this minor improvement of electrical conductivity can still be employed to pattern electrical properties along preferred directions in deposited structures. A simple rectangular hydrogel construct with patterned electrical properties was printed by using the combination of A2, AC1, AC3 and AC5 inks (Fig. 5a). A gradient pattern was deployed in the printed object in which specific conductivity increased gradually from one end to another. Matrix composed of A2 ink had a very low conductivity compared to the patterned segments of the object in the same ambient environment. In this prospect, each segment in deposited filaments can act as an independent resistor and contribute to the overall conductivity of the printed object. To validate this concept, series of single filaments with fixed lengths composed of AC1 and AC3 inks and with three different structure designs were printed, while the length, and hence the volume, of each ink segment varied among different designs. Figure 5c shows the values of specific conductivity in single filaments of each structure design. Specific conductivity of filaments was between those values of AC1 and AC3 inks, and by increasing the length of less conductive region (AC1), the overall conductivity decreased as was expected. If the configuration of segments in each design is considered as resistors in a series circuit, a deviation from expected overall conductivity of single filaments was observed for all three designs. This deviation from expected values might be attributed to the effect of interfaces in assembling of different segments.

**Figure 6:**
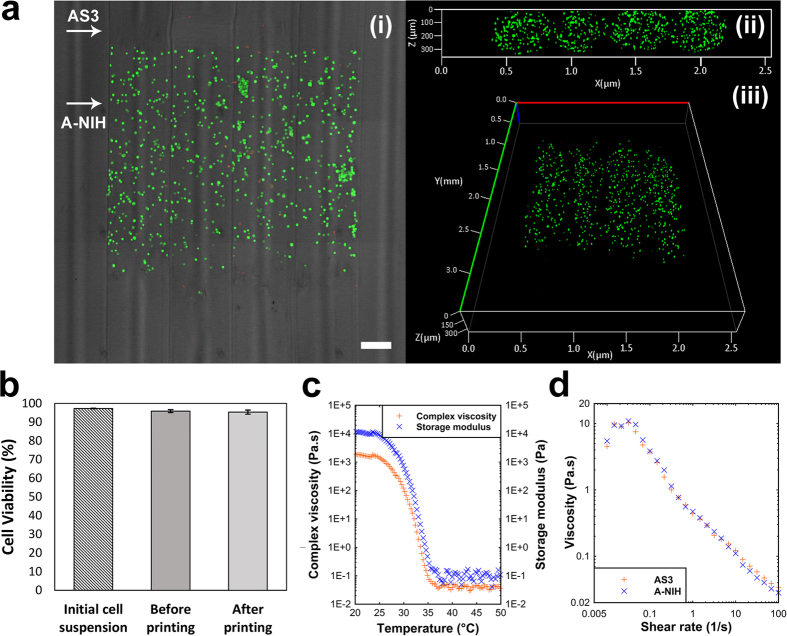
Patterning cell encapsulated regions within a printed object. (**a**)(i–iii) Confocal images of a sample with patterned encapsulated NIH 3T3 cells inside. (i) Top view of the projected z stacks on XY plane combined with phase contract image; scale bar, 250 μm. (ii,iii) Fluorescent images of cross section and perspective views of the sample. (**b**) Results of cell viability assay at Day 0 prior and after printing compared to control sample. (**c**) Viscosity and storage modulus of AS3 ink as a function of temperature. Gelation was determined by G′-G′′ crossover point at 35 °C and viscosity of ink increased significantly by decreasing temperature. Ink has shown liquid like behavior while it was kept at 38 °C in ink’s reservoir. (**d**) Shear thinning behavior of AS3 and A-NIH inks at 38 °C. Viscosity dropped significantly by increasing shear rate.

To demonstrate the ability of our method in deposition of heterogeneous biologically functional patterns, a combination of A-NIH and AS3 inks were used to produce a simple structure patterned with NIH-3T3 cells inside an agarose matrix. Temperature at which the bio-ink and AS3 ink were kept during printing was set to 38 °C to prevent from gelation of inks and in the meantime, keep the encapsulated cells alive. Figure 6a demonstrates distribution of cells across the sample (top view, cross-sectional view, and perspective view). A sharp interface between cell laden and cell free segments of filaments is evident while no structural defect is visible at the interface section. Results of cell viability are shown in Fig. 6b. Cell viability did not change significantly prior and after printing compared to the starting cell pellets before preparation of A-NIH bio-ink. Comparison between viability of cells prior and after printing suggests that the printing process does not induce damage to cells. Referring to rheological data provided in Fig. 6c,d, one can see that low gelation temperature of AS3 ink (35 °C as determined by G’-G” cross-over point) and shear thinning behavior of inks have reduced the risk of damage to cells caused by high shear rates during aspiration. The Power Law (Oswald) model was used to quantify shear thinning behavior of two inks. Flow index values for both AS3 and A-NIH inks (n = 0.3 and 0.26, respectively) and the trend of viscosity-shear rate profiles were almost the same, although A-NIH bio-ink showed less non-Newtonian behavior in lower shear rates.

### 3-Dimentional deposition of patterned structures

**Figure 7:**
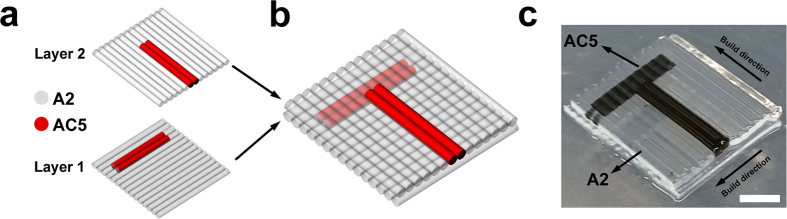
3D printing of a patterned hydrogel structure. (**a**) Schematic representation of two single layers each composed of a pattern including A2 and AC5 inks. (**b**) CAD model of the 3D patterned object. Combination of AC5 inks in two layers resembled a “T” shape pattern in 3D. (**c**) 3D printed patterned hydrogel structure; scale bar, 2 mm.

By using the possibility of creating patterns in each deposited layer, a 3D object was printed which is shown in Fig. 7. The structure was composed of two layers that each were sequentially deposited with an individual pattern (Fig. 7a), and together both layers resembled a “T” shape pattern (Fig. 7b). Printing parameters (i.e. process and material related ones) were controlled in the same manner they have been manipulated in deposition of single layers of patterned structures.

## Discussion

We have demonstrated the ability of our proposed method in producing heterogeneous 3D printed hydrogel structures. The significance of this new method besides its simple nature is the capability of manipulating 3D printing parameters to yield a broad range of functionalities in deposited object and tuning different properties of starting inks to obtain the desired features. Mutual dependency of effective process parameters (such as temperature, aspiration rate, and printer head speed) and physical properties of inks is evident by considering the effect of temperature on flow behavior of inks, i.e. viscosity-temperature profiles. A temperature framework could be established based on inks’ composition to keep the starting materials at desired viscosities and also control the heat loss during ink switching. Hydrogel viscosity plays a crucial role in determination of printing fidelity since it implicates the resistance of fluid to flow under applied stress^16^. The design of our dispensing system and the employed deposition mechanism in which the ink(s) turn to gel from liquid state inside the glass capillary, guaranties shape accuracy of deposited filaments since they are already viscous enough to retain their shape during deposition. By keeping the ink’s reservoir temperature constant, ink viscosity variations could also be minimized during the aspiration. This means that applied shear stress can be controlled, which is critical for aspiration of sequenced segments and prevention of generating non-homogeneous mixture of inks at the switching interface. Moreover, applied stress can be monitored by controlling viscosity in shear-stress sensitive inks and depending on the range of available process temperatures, the output functionality of ink can be maximized. The viscosity of cell-laden hydrogels should be controlled to optimize the applied pressure on encapsulated cells and maximize the viability in an extrusion based deposition system^17^,^18^,^19^,^20^.

Depending on the desired output functionalities, material selection should be performed by considering a set of factors, flow characteristics, the possibility of tuning structural parameters, and also meeting specific criteria depending on the desired applications like bioprinting, just to name a few. Agarose is a polysaccharide with tunable flow behavior and stiffness^21^. Moreover, the excellent printing quality of agarose made it a reasonable choice for demonstration of the potentials of our proposed method^16^, although there are some concerns about the bioactivity of agarose hydrogels^22^,^23^. If the biofunctionality of the hydrogel in a heterogeneous structure is likely to be examined, a more bioactive hydrogel should be considered as the candidate material for bioprinting purposes^24^.

**Figure 8:**
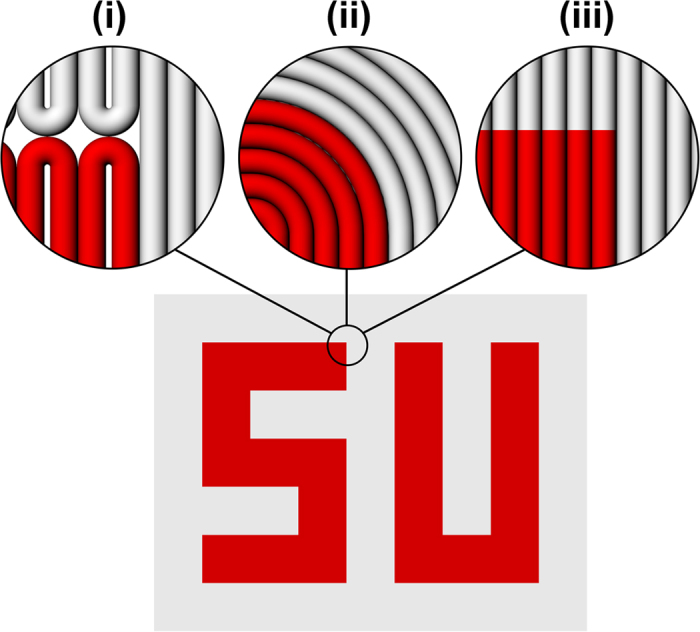
Schematic representation of the effect of changing printing tool-path parameters on resolution of printed object. Comparison between (**i,ii**) two typical continuous tool-paths, and (**iii**) our proposed method in printing of a sharp-edged corner.

Preserving the dimensional accuracy of deposited pattern in hydrogel constructs is evident in the printed samples (Figs 1, 4, 5, 6 and 7). Current methods of multi-material hydrogel deposition in extrusion based approaches lack the necessary geometrical precision in preserving features of CAD models at sharp-edged regions. Even by optimizing the tool-path, sharp edges are usually rounded off in continuous deposition of filaments. Figure 8 schematically represents some possible tool-paths to produce a typical edge in aforementioned SU pattern, including our proposed method. Curvature at the edges in conventional tool-path designs (Fig. 8(i,ii)) can be reduced by reducing filament diameter although there exist certain limits beyond which printing will be practically impossible. Depending on the nozzle geometry and dispensing mechanism, printing time and shear stress at nozzle tip are directly affected by decreasing nozzle diameter^20^,^25^ which might negatively affect print quality. Moreover, a quantitative study^26^ on deposition parameters in the case of aqueous hydrogels suggested that decreasing nozzle diameter will not necessarily result in improved resolution. As stated before, applied shear stress induced by smaller nozzle diameters should also be monitored carefully to avoid undesired changes in inks’ properties, especially for bio-inks used in bioprinting practices^17^,^18^,^19^,^20^. From geometrical point of view, an edge can be defined as the interface between two neighboring segments with different properties. As we demonstrated before, integrating multiple inks in a single filament results in enhanced sharpness at the edges of patterned segments (schematically presented in Fig. 8(iii)). However, this enhancement substantially depends on the pattern’s geometry. Patterns which are defined as curved objects or consist of non-perpendicular edges (outline edges of patterned area itself, or the angle between extended outlines of patterned area and those of background/matrix) will turn to be as jaggy segments. The resolution of the pattern will depend on filament diameter; the smaller the filament diameter the higher resolution at the curved edges, although the technical limits in reaching smallest possible diameters will eventually determine the resolution of pattern. The aforementioned geometrically dependent limitation is considered as a challenge in current robotic dispensing based printing practices both in continuous and discrete deposition methods. Compared to conventional deposition system designs, the proposed method has the advantage of improving pattern resolution while retaining structural integrity of neighboring segments by merging multiple segments into a single deposited filament. Moreover, changing segmentation orientation is an alternative approach to minimize the number and length of jagged edges in the case of non-perpendicular pattern’s edges.

Recently, Hardin *et al*.^11^ developed a microfluidic printhead for continuous direct ink writing of heterogeneous structures based on viscoelastic inks. By employing their new design, they were able to print 1D, 2D and 3D heterogeneous structures with good control over transition sharpness of subsequent segments in printed object. Although their design was able to seamlessly switch between printing two different materials, it is strictly limited to viscoelastic inks. Viscosity played a crucial role in controlling the interface of transition and by decreasing the viscosity, its sharpness would probably decrease. Hydrogels, while they are in the liquid like form, have very low viscosity which makes them unsuitable to be processed by such nozzle designs to yield high dimensional accuracy and also acceptable print fidelity. However, tuning flow behavior by altering the chemistry^27^ might be a possible option to extend the application of such printhead designs to less viscous materials. In contrast, our approach in assembling hydrogel segments with different compositions and functionalities inside the glass capillary provides the opportunity to control transition sharpness precisely and effectively in processing of inks with low viscosity.

Common multi-nozzle approaches in 3D printing of heterogeneous hydrogel structures rely on deposition of each ink at the starting/ending point of previously deposited material. This feature will result in inaccuracy of deposition as well as inducing gaps or in a general term structural defects, at the patterned segments’ interface with the matrix. The capability of our method in producing sharp-edged and at the same time highly integrated segments, provides the opportunity to selectively and continuously switch between different functionalities/properties in deposited structure. Structural integrity of two neighboring segments in each filament (Fig. 3c–e) is especially important when the printed object is intended to have patterned mechanical properties or to be used under mechanical stress. The integrity of interface will improve load transfer along two segments which in turn will enhance the stability of object under mechanical loading. Moreover, the ability to create patterned hydrogel structures with different mechanical properties and high dimensional accuracy showed great promise for applications in the field of tissue engineering since the stiffness of scaffold/substrate can affect cell-biomaterial response and direct cell spreading^28^,^29^,^30^, migration^31^ and differentiation^28^,^30^,^32^.

Incorporation of carbon nanostructures like carbon nanotubes, graphene, or other electrically conductive nanoparticles in a hydrogel matrix results in hydrogel nanocomposites with enhanced electrical properties and emerges new potentials and functionalities^33^,^34^. Patterning electrical properties with high dimensional accuracy is favorable in a wide range of applications such as sensors, conductive substrates, actuators and tissue engineering constructs. As we demonstrated in the previous section, we were able to deposit electrically conductive patterns by altering the composition of hydrogel inks. We speculate that geometrically dependent electrical conductivity of patterned sections can be further used in combination with the ability to selectively pattern encapsulated cells in hydrogel matrices, which will result in selective improvement of cell signaling^35^,^36^. Furthermore, we have shown that how the geometry of each individual segment, which possesses its own electrical properties, can contribute to the overall conductivity of patterned object. The flexibility in rearrangement of individual segments in the designed structure would be of great interest, especially when there is a need for placement of certain geometrically dependent electrical conductivity or resistivity patterns, and in the same time meet the bulk design criteria.

By increasing the complexity of CAD models especially in the case of anatomically mimicked structures in tissue engineering, intricacy of patterns will grow and features like hangover geometries will appear in CAD models which need to be supported temporarily. Use of support structures were reported in a number of studies^37^,^38^,^39^ aimed at fabrication of tubular 3D constructs with hangover geometries. Depending on the final geometry and functionality of printed object, developing a self-supporting structure with good structural integrity is desirable. As we demonstrated in the previous section, our method is capable of producing biologically active patterns in a hydrogel matrix (Fig. 6a). The bioprinted constructs could have several multi-functionality. Figure 6 shows six filaments, four of them composed of three segments with different functions. Figure 6a shows a hydrogel object which was deposited with patterned functionalities by using AS3 ink as the support structure and A-NIH ink as the carrier for the encapsulated cells. Good stability of the interface in printed objects will result in enhanced mechanical durability of construct which is a critical issue in fabrication of large objects. Combination of structural integrity and patterned encapsulated cell densities provides new opportunities in the development of self-supporting hydrogel constructs for regenerative tissue engineering applications.

The length of the size of the micro-capillary glass tube used in the printing system determines length of a single filament which can be printed in a single extrusion. In the current 3D printer system, a micro capillary glass tube with a length of 85 mm and 500 μm inner diameter is used. Theses parameters are used as an input parameter for the developed CAD-based algorithm. Based on the CAD design, one can fabricate structures with or without gaps between single filaments and this parameter is strictly related to the design criteria. The number of bioink segments can be printed depends on the length of the glass capillary and the degree of similarity of flow behaviors of different inks. The resolution of the printed structures is limited to the geometrical resolution (diameter of a single filament and consequently the layer by layer resolution of printed object) and the resolution of patterning. The former can be manipulated by modification of hardware (i.e. diameter of glass tube and its length), while the later can be enhanced by modification of materials properties (i.e. the viscosity which will directly affect the flow behavior, and hence the minimum length of a region which is possible to aspirate without extensive mixing at the interface).

## Methods

### Preparation and characterization of inks and printed hydrogels

Agarose inks (A1 to A5) were prepared by dissolving agarose powder (A9539, Sigma-Aldrich) in distilled water by using boiling water bath method provided by the producer. AS3 ink was prepared by autoclaving the mixture of low melting temperature agarose (Biozym Sieve 3:1) and 1X phosphate buffer saline (PBS, Hyclone by Thermo Scientific) for 2 hours. Aqueous suspension of 0.1 wt% single-wall carbon nanotubes (SWCNT) with 0.1 wt% Polyvinylpyrrolidone (PVP) supplied by OSCiAl was used to produce SWCNT embedded nanocomposite hydrogels. Adequate amounts of agarose powder were added to aqueous dispersions of SWCNTs kept in a boiling water bath and stirred vigorously for 30 minutes to completely melt agarose powders. Mixtures were sonicated for 30 minutes using a probe sonicator (Q700, QSonica) while the amplitude and pulse on/off interval were set to 50% and 5 seconds, respectively. Temperature was maintained at 80 °C during sonication. NIH 3T3 cells were used to prepare cell laden bio-ink. Cells were cultured in Dulbecco’s Modified Eagle Medium (DMEM, Sigma) containing 10% fetal bovine serum (FBS, Sigma) and 1% penicillin-streptomycin (Gibco), passaged two times a week and incubated in humidified atmosphere containing 5% CO_2_ at 37 °C. To prepare cell laden bio-ink, cell cultures were washed with 1X PBS and trypsinized for 5 min followed by centrifuging at 1100 rpm for 5 min. Cell pellets were re-suspended in growth medium and counted with hemocytometer after staining with trypan blue (Sigma). The proper amount of cells was added to AS3 ink kept at 38 °C to obtain cell laden bio-ink with 1 × 10^6^ cells/ml density.

Rheological properties of inks were investigated using Anton Paar MCR 302 rheometer (Anton Paar, Austria) using 25 mm diameter parallel plates configuration. Samples were subjected to oscillatory shear stress (strain = 1%, frequency = 1 Hz) during cooling down from 50 to 20 °C with 6 °C/min ramp, and complex shear modulus and complex viscosity of samples were recorded. Rotational rheometry was used by applying a shear rate sweep from 0.01 to 100 (1/s) at 38 °C for AS3 and A-NIH inks. Angular frequency sweep from 0.1 to 10 rad/s was applied at 38 °C to determine network stability of AS3 and A-NIH inks.

Cylindrical gel samples with 25 mm diameter and 30 mm height from A1 to A5 inks were prepared to perform uniaxial compression testing with the designed experimental setup. Experimental setup included a dynamometer (Kistler 9256C1) placed on the lower plate which was interfaced with data acquisition software (LabVIEW, National Instruments) to log the measured force during compression. The upper plate was connected to a high resolution stepper motor. To minimize the effect of friction on the contact plates and prevent from sample barreling during compression, a water repellant silicon compound lubricant (Dow Corning) was used^40^. A prestrain of 1% with the head speed of 1 mm/min was applied prior starting the measurements. Five samples of each ink formulation were uniaxially compressed while the maximum strain was confined to 15% and the compression rate was set to 2 mm/min. Compressive loads versus upper plate displacement were recorded for each sample and stress-strain plots were generated accordingly. Small strain portion of stress-strain plots (up to 4% strain) were considered as the linear segment of plots at which the samples were described by the Hook’s law. In order to qualitatively show the heterogeneity of mechanical properties of the printed objects, a method based on observation of apparent deformation in the printed object was conceived. Series of parallel filaments with the same lengths were printed with two segments each, keeping the same segments of filaments at the same side of the deposition plane with respect to the central interface sections. A Pi (Π) shape bar was designed and 3D printed by using Amber 3D printer (Autodesk, USA) which weighed 5.37 g. The bar was placed just above the printed filaments, one leg over each set of segments, and then it was released upon touching the filaments and let to be sunk due to its own weight. The amount of deformation induced by the penetration of legs was recorded as timed sequences of images.

To measure the electrical properties of gels, single filaments of each corresponding ink were printed on a polystyrene petri dish and droplets of gallium/indium eutectic (Sigma-Aldrich) were placed on each end of filaments. Agilent U1273A handheld digital multimeter connected to Cascade PM5 Port Probe Station was used to measure the electrical resistance of each filament. Resistance values were recorded for 15 seconds in each measurement. Five samples were measured from each hydrogel ink and the mean values were reported. Specific conductivity of filaments with different configurations of constitutive segments were measured with the same method. Three different configurations of AC1 and AC3 inks were designed (namely, design 1, design 2, and design 3). For each design the printed filaments of 24 mm size were composed of AC3-AC1-AC3 segments, while the length of AC1 (middle) segment in each design was 4, 8, and 12 mm, respectively. In the same way, the length of AC3 heads and tails in the printed filaments of each design were equal to each other as 10, 8, and 6 mm, respectively.

### Printing hydrogel structures

We developed a custom-built 3D printer consists of a three-axis motion control stage with servo motors in each axis and a plunger based dispensing unit motorized by a high resolution stepper motor. Machine was controlled by a personal computer through Mach3 control system (Newfangled Solutions, USA). The printer was equipped with a temperature controlled heating/cooling unit which was designed to maintain inks at a desired temperature during printing. The deposition speed of filaments was set to 150 mm/min while the aspiration/extrusion speed of inks varied over 50 to 100 mm/min. The computer aided designs were developed using Rhino software (Robert McNeel & Associates) and the tool-paths were generated by using Rhinoscript. Aspiration-on-demand was conducted through the execution of a set of algorithms and protocols, beginning with layer by layer segmentation of CAD models to assign the aspiration orders. Each two neighboring segments of a single multimaterial filament in the CAD model reassembled one aspiration step. The developed code calculates each segment of multimaterial filaments directly from computer models and determines aspiration sequence and the amount of aspiration for each ink segment automatically. Deposition path plan is then determined based on the computer model. One advantage of using the glass capillary tube as the ink carrier is its fixed internal diameter, so the aspiration volume would be easily calculated and automatically set in the generated path plan only by using the length of each.

### Imaging of printed structures

The studies on interface between different segments in printed filaments were done by using Carl-Zeiss LSM 710 inverted confocal microscope. Fluorescent microbeads with excitation wavelength of 405 nm (Createx Colors, USA) were used to selectively mark segments in each filament. To visualize the patterned segments in printed constructs including bio-ink, and also to quantify the viability of cells prior and after printing, the following cell staining protocols were fallowed. Cell viability prior printing (in cell suspension) was evaluated by using a trypan blue exclusion test and cells were visually examined and scored. Cell viability in cell laden bio-ink kept in ink’s reservoir and within printed patterned constructs was assessed by applying a live/dead fluorescence assay just after printing. Printed constructs were first stained by incubating in 2 μM calcein-AM (Invitrogen, green fluorescence) and 0.5 μM propidium iodide (Invitrogen, red fluorescence) for 20 min at 37 °C, followed by washing by 1X PBS for three times. Tiled z stacks were then captured by Carl-Zeiss LSM 710 inverted confocal microscope and the 3D reconstructed images were quantitatively analyzed by ImageJ software^41^. The 3D Object Counter plug-in for ImageJ was used to quantify the number of live/dead cells in 3D stacks. All the patterned samples were directly printed, stained and washed on the same glass-bottom petri dishes. To capture real-time images and videos during deposition of filaments a NX4-S2 high speed camera (Integrated Design Tools, IDT) coupled with the Infinity K2 DistaMax macro lens (Infinity Photo-Optical) was used and data were recorded and processed by Motion Studio software suite (Integrated Design Tools, IDT).

## Additional Information

**How to cite this article**: Nadernezhad, A. *et al*. Multifunctional 3D printing of heterogeneous hydrogel structures. *Sci. Rep*. **6**, 33178; doi: 10.1038/srep33178 (2016).

## Supplementary Material

Supplementary Video S1

Supplementary Video S2

Supplementary Video S3

## Figures and Tables

**Table 1:** Ink formulations used in deposition of patterned constructs.

Ink	Agarose concentration (w/v%)	SWCNT concentration (w/v%)	NIH 3T3 cell concentration (cells mL^−1^)
A1	1	0	0
A2	2	0	0
A3	3	0	0
A4	4	0	0
A5	5	0	0
AC1	2	0.01	0
AC2	2	0.02	0
AC3	2	0.03	0
AC4	2	0.04	0
AC5	2	0.05	0
AS3*	3	0	0
A-NIH*	3	0	1×10^6^

^*^Low melting temperature agarose (sieve) was used as the primary hydrogel ink.

## References

[b1] Parekh D. P., Cormier D. & Dickey M. D. Multifunctional Printing: Incorporating Electronics into 3D Parts Made by Additive Manufacturing. Additive Manufacturing 215 (2015).

[b2] Ahn B. Y. . Omnidirectional printing of flexible, stretchable, and spanning silver microelectrodes. Science 323, 1590–1593 (2009). 19213878 10.1126/science.1168375

[b3] Kong Y. L. . 3D Printed Quantum Dot Light-Emitting Diodes. Nano letters 14, 7017–7023 (2014). 25360485 10.1021/nl5033292

[b4] KANG H., Kengla C., Lee S., Yoo J. & Atala A. 3-D organ printing technologies for tissue engineering applications. Rapid Prototyping of Biomaterials: Principles and Applications 236 (2014).

[b5] Snyder J. E. . Combined multi-nozzle deposition and freeze casting process to superimpose two porous networks for hierarchical three-dimensional microenvironment. Biofabrication 6, 015007 (2014). 24429830 10.1088/1758-5082/6/1/015007

[b6] Yan F. . A multi-scale controlled tissue engineering scaffold prepared by 3D printing and NFES technology. AIP Advances 4, 031321 (2014).

[b7] Inzana J. A. . 3D printing of composite calcium phosphate and collagen scaffolds for bone regeneration. Biomaterials 35, 4026–4034 (2014). 24529628 10.1016/j.biomaterials.2014.01.064 PMC4065717

[b8] Kolesky D. B. . 3D bioprinting of vascularized, heterogeneous cell‐laden tissue constructs. Advanced Materials 26, 3124–3130 (2014). 24550124 10.1002/adma.201305506

[b9] Bertassoni L. E. . Direct-write bioprinting of cell-laden methacrylated gelatin hydrogels. Biofabrication 6, 024105, doi: 10.1088/1758-5082/6/2/024105 (2014). 24695367 PMC4040163

[b10] Jafari M. . A novel system for fused deposition of advanced multiple ceramics. Rapid Prototyping Journal 6, 161–175 (2000).

[b11] Hardin J. O., Ober T. J., Valentine A. D. & Lewis J. A. Microfluidic Printheads for Multimaterial 3D Printing of Viscoelastic Inks. Advanced Materials (2015). 10.1002/adma.201500222 25885762

[b12] Chimate C. & Koc B. Pressure assisted multi-syringe single nozzle deposition system for manufacturing of heterogeneous tissue scaffolds. The International Journal of Advanced Manufacturing Technology 75, 317–330 (2014).

[b13] Jungst T., Smolan W., Schacht K., Scheibel T. & Groll J. R. Strategies and Molecular Design Criteria for 3D Printable Hydrogels. Chemical reviews (2015). 10.1021/acs.chemrev.5b00303 26492834

[b14] Manickam K., Machireddy R. R. & Seshadri S. Characterization of biomechanical properties of agar based tissue mimicking phantoms for ultrasound stiffness imaging techniques. Journal of the mechanical behavior of Biomedical Materials 35, 132–143 (2014). 24769915 10.1016/j.jmbbm.2014.03.017

[b15] Lewitus D. Y. . Biohybrid carbon nanotube/agarose fibers for neural tissue engineering. Advanced functional materials 21, 2624–2632 (2011). 21887125 10.1002/adfm.201002429 PMC3163387

[b16] Malda J. . 25th anniversary article: engineering hydrogels for biofabrication. Advanced Materials 25, 5011–5028 (2013). 24038336 10.1002/adma.201302042

[b17] Cheng J. . Rheological properties of cell-hydrogel composites extruding through small-diameter tips. Journal of Manufacturing Science and Engineering 130, 021014 (2008).

[b18] Aguado B. A., Mulyasasmita W., Su J., Lampe K. J. & Heilshorn S. C. Improving viability of stem cells during syringe needle flow through the design of hydrogel cell carriers. Tissue Engineering Part A 18, 806–815 (2011). 22011213 10.1089/ten.tea.2011.0391 PMC3313609

[b19] Kong H. J., Smith M. K. & Mooney D. J. Designing alginate hydrogels to maintain viability of immobilized cells. Biomaterials 24, 4023–4029 (2003). 12834597 10.1016/s0142-9612(03)00295-3

[b20] Li M., Tian X., Schreyer D. J. & Chen X. Effect of needle geometry on flow rate and cell damage in the dispensing‐based biofabrication process. Biotechnology progress 27, 1777–1784 (2011). 22238771 10.1002/btpr.679

[b21] Normand V., Lootens D. L., Amici E., Plucknett K. P. & Aymard P. New insight into agarose gel mechanical properties. Biomacromolecules 1, 730–738 (2000). 11710204 10.1021/bm005583j

[b22] Kao J. M., Rose R., Yousef M., Hunter S. K. & Rodgers V. *In vivo* biocompatibility evaluation of Cibacron blue-agarose. Journal of biomedical materials research 47, 537–542 (1999). 10497289 10.1002/(sici)1097-4636(19991215)47:4<537::aid-jbm10>3.0.co;2-i

[b23] Oliveira J. T. & Reis R. Polysaccharide‐based materials for cartilage tissue engineering applications. Journal of tissue engineering and regenerative medicine 5, 421–436 (2011). 10.1002/term.335 20740689

[b24] Annabi N. . 25th anniversary article: rational design and applications of hydrogels in regenerative medicine. Advanced Materials 26, 85–124 (2014). 24741694 10.1002/adma.201303233 PMC3925010

[b25] Barry R. A. . Direct‐Write Assembly of 3D Hydrogel Scaffolds for Guided Cell Growth. Advanced materials 21, 2407–2410 (2009).

[b26] Kang K., Hockaday L. & Butcher J. Quantitative optimization of solid freeform deposition of aqueous hydrogels. Biofabrication 5, 035001 (2013). 10.1088/1758-5082/5/3/035001 23636927

[b27] Rutz A. L., Hyland K. E., Jakus A. E., Burghardt W. R. & Shah R. N. A Multimaterial Bioink Method for 3D Printing Tunable, Cell‐Compatible Hydrogels. Advanced Materials 27, 1607–1614 (2015). 25641220 10.1002/adma.201405076 PMC4476973

[b28] Kloxin A. M., Kloxin C. J., Bowman C. N. & Anseth K. S. Mechanical properties of cellularly responsive hydrogels and their experimental determination. Advanced materials 22, 3484–3494 (2010). 20473984 10.1002/adma.200904179 PMC3890982

[b29] Nikkhah M., Edalat F., Manoucheri S. & Khademhosseini A. Engineering microscale topographies to control the cell–substrate interface. Biomaterials 33, 5230–5246 (2012). 22521491 10.1016/j.biomaterials.2012.03.079 PMC3619386

[b30] Marklein R. A. & Burdick J. A. Spatially controlled hydrogel mechanics to modulate stem cell interactions. Soft Matter 6, 136–143 (2010).

[b31] Tayalia P., Mendonca C. R., Baldacchini T., Mooney D. J. & Mazur E. 3D Cell‐Migration Studies using Two‐Photon Engineered Polymer Scaffolds. Advanced Materials 20, 4494–4498 (2008).

[b32] Engler A. J., Sen S., Sweeney H. L. & Discher D. E. Matrix elasticity directs stem cell lineage specification. Cell 126, 677–689 (2006). 16923388 10.1016/j.cell.2006.06.044

[b33] Kuilla T. . Recent advances in graphene based polymer composites. Progress in polymer science 35, 1350–1375 (2010).

[b34] Goenka S., Sant V. & Sant S. Graphene-based nanomaterials for drug delivery and tissue engineering. Journal of Controlled Release 173, 75–88 (2014). 24161530 10.1016/j.jconrel.2013.10.017

[b35] Shin S. R. . Carbon-nanotube-embedded hydrogel sheets for engineering cardiac constructs and bioactuators. ACS nano 7, 2369–2380 (2013). 23363247 10.1021/nn305559j PMC3609875

[b36] Shin S. R. . Carbon nanotube reinforced hybrid microgels as scaffold materials for cell encapsulation. ACS nano 6, 362–372 (2011). 22117858 10.1021/nn203711s PMC3401631

[b37] Visser J. . Biofabrication of multi-material anatomically shaped tissue constructs. Biofabrication 5, 035007 (2013). 23817739 10.1088/1758-5082/5/3/035007

[b38] Skardal A., Zhang J. & Prestwich G. D. Bioprinting vessel-like constructs using hyaluronan hydrogels crosslinked with tetrahedral polyethylene glycol tetracrylates. Biomaterials 31, 6173–6181 (2010). 20546891 10.1016/j.biomaterials.2010.04.045

[b39] Kucukgul C. . 3D bioprinting of biomimetic aortic vascular constructs with self‐supporting cells. Biotechnology and bioengineering 112, 811–821 (2015). 25384685 10.1002/bit.25493

[b40] Zhao W. . A methodology to analyse and simulate mechanical characteristics of poly (2‐hydroxyethyl methacrylate) hydrogel. Polymer International 62, 1059–1067 (2013).

[b41] Schneider C. A., Rasband W. S. & Eliceiri K. W. NIH Image to ImageJ: 25 years of image analysis. Nature methods 9, 671–675 (2012). 22930834 10.1038/nmeth.2089 PMC5554542

